# Traumatic Brain Injury: Unmet Support Needs of Caregivers and Families in Florida

**DOI:** 10.1371/journal.pone.0082896

**Published:** 2013-12-17

**Authors:** Christina Dillahunt-Aspillaga, Tammy Jorgensen-Smith, Sarah Ehlke, Melanie Sosinski, Douglas Monroe, Jennifer Thor

**Affiliations:** 1 Department of Rehabilitation and Mental Health Counseling, University of South Florida, Tampa, Florida, United States of America; 2 Department of Anthropology, University of Florida, Gainesville, Florida, United States of America; 3 Department of Psychology, University of North Carolina Wilmington, Wilmington, North Carolina, United States of America; 4 Department of Social Work, University of South Florida, Tampa, Florida, United States of America; 5 Brain Injury Association of Florida, Inc., Tallahassee, Florida, United States of America; 6 WellFlorida Council, Inc., Gainesville, Florida, United States of America; George Mason University/Krasnow Institute for Advanced Study, United States of America

## Abstract

Sustaining a Traumatic Brain Injury results in familial strain due to the significant impact the injury has upon the role and function of individuals and their families at home and in the community. Using the Stress Process Model of Caregiving, a caregiver needs assessment survey was developed and conducted to better understand the needs of individuals with a Traumatic Brain Injury and their caregivers. Survey results indicate that caregivers experience many challenges including unmet needs in areas of relational supports such as maintaining relationships, long-term emotional and financial support for themselves and the survivor, and the need for a patient or caregiver advocate. Implications for future practice are presented.

## Introduction

Traumatic brain injury (TBI) is a common multifaceted injury that occurs when there is damage to the brain caused by an external force, such as a fall, motor-vehicle accident, gun-shot to the head, other form of assault, or sports-related injury. TBI can affect all aspects of functioning including basic activities of everyday life such as personal care, ambulation, mobility, and higher-level psychosocial functioning (e.g.: employment, social relationships, independent living, and recreation) [1]. Nationally, there are 5.3 million Americans living with a long-term disability resulting from a TBI. According to the Centers for Disease Control, 1.7 million people sustain a TBI each year [2]. Researchers estimate that 3.2 million Americans need long-term and life-long assistance to perform daily life activities as a result of sustaining a TBI [3]. Individuals with TBI often rely on caregivers for long-term and life-long assistance. While there is abundant literature addressing the needs of survivors of TBI, research focusing on the needs of caregivers is less developed and warrants further investigation [7], [25]. This article explores needs identified by caregivers of individuals with TBI in order to better understand the barriers and challenges experienced.

### Review of the Literature

The cost of care for individuals with TBI is significant with lifetime costs of care averaging around 4 million dollars [4]. In 2000, direct medical costs and indirect costs (e.g.: lost productivity) of TBI totaled an estimated 76.5 billion dollars in the United States [5]. After a person sustains a TBI, commonly they will require acute inpatient rehabilitation which lasts approximately two months. Additional inpatient rehabilitation options are available, but are expensive and average around 1,000 dollars per day [4]. Therefore, many individuals with mild to moderate TBI return home to live with their families shortly after sustaining their injury and thus, the family becomes the primary caregiver. Research has shown that family members are often responsible for providing financially for the person with injury and for facilitating social needs including leisure activities and community outings [6].

#### Caregiving and Family Needs

For purposes of this article, a caregiver is defined as an unpaid individual who provides care services to those who cannot adequately care for themselves [4]. Caregivers and families play an important, all-encompassing role, in the rehabilitation process of individuals with TBI. This role is often accompanied by an overwhelming sense of stress that is experienced by the individual with TBI and their entire family [8]. Studies have shown that caregivers of individuals with TBI experience chronically high levels of distress when compared to caregivers of other populations, such as caregivers of people with intellectual disabilities [16], [25], [32]. Stress results from a variety of aspects of caregiving including neurobehavioral and mood disturbances associated with the injury, the overall demands of caring for the individual with TBI, lack of appropriate social supports, limited access to important resources and services, and changes within the family structure. Behavioral and cognitive problems are a common consequence of TBI. These challenges interact in complex ways and usually require integrated and multifaceted interventions and support [31]. Previous research indicates that the presence and extent of neurobehavioral challenges seems to predict caregiver burden more strongly than severity of injury, physical impairments, and even cognitive impairments [9]. Despite limitations associated with physical or cognitive impairments, the existing literature suggests that neurobehavioral symptoms tend to be the most distressing symptoms for the family and are more strongly related to poor outcomes for the patient and their family [10]. Yet, critical care settings rarely frame the treatment of individuals with TBI as neurobehavioral [11].

Research assessing long-term family needs of caregivers of persons with TBI indicates that many needs, including information and support, are frequently rated as unmet [9]. Caregiver needs such as health and rehabilitation information, financial advice and assistance, and emotional and social support change over time and reflect the care setting, the stage in recovery (from acute-care to long-term living in the community), level of functioning and personal roles of the person with TBI [12]. The evolution of caregiver needs reflects the chronic and evolving disease process of a TBI that often negatively impacts the lives of people involved [13]. Therefore, caregiver resources that fail to accommodate the dynamic disease process of TBI may fall short in adapting to caregiver needs.

The lack of adequate services and supports available for caregivers and their families places them at an increased risk of depression, frustration, anxiety, and burden [7], [14], [15], [16], [30]. Burden is defined as the distress that a family member/caregiver experiences as a result of the changes that they observe in the person with TBI [14], [33]. The sequelae of TBI and limited access to resources, in combination with the prolonged distress and physical demands of caregiving, have demonstrated that caregiving is an independent risk factor for mortality [23]. According to previous research, the caregiver's well-being affects both their capacity and willingness to care for the injured person; and thus impacts the overall quality of life for both the person with TBI and the remaining family members [8], [17], [18], [24]. Therefore, caregivers in better health may impart some protective effect on the health of the recipient of care. Interventions that are tailored to promote family well-being may influence the health and quality of life for the individual with TBI in functional, measurable ways. Intervention program components should include information about the sequelae, or injury-related negative after-effects of TBI, as well as components to assist caregivers in developing problem-solving strategies to address difficulties within the home, which appear to be an essential aspect of reducing caregiver burden [8], [19].

#### Stress Process Model of Caregiving

Studies of factors relevant to the caregiving experience often lack consistent measures and a strong theoretical framework due to their global and diverse nature [15]. The lack of conceptual clarity in this area of study has resulted in the use of various terminologies pertaining to caregiving, with more than 50 different instruments used across studies [20]. While most researchers measuring caregiver stress focus on caregiver burden, (which looks at stress directly related to caregiving), the Stress Process Model of Caregiving for Individuals with TBI (SPMC) examines both positive and negative caregiving experiences. This process model provides an opportunity to explore relationship strengths as well as obstacles [15]. In turn, the SPMC examines caregiver resiliency and potential for empowerment within the context of their obstacles. By understanding what the caregiver perceives as their strengths and resources, it is then possible to empower caregivers to utilize these internal and external supports [16].

**Figure 1:**
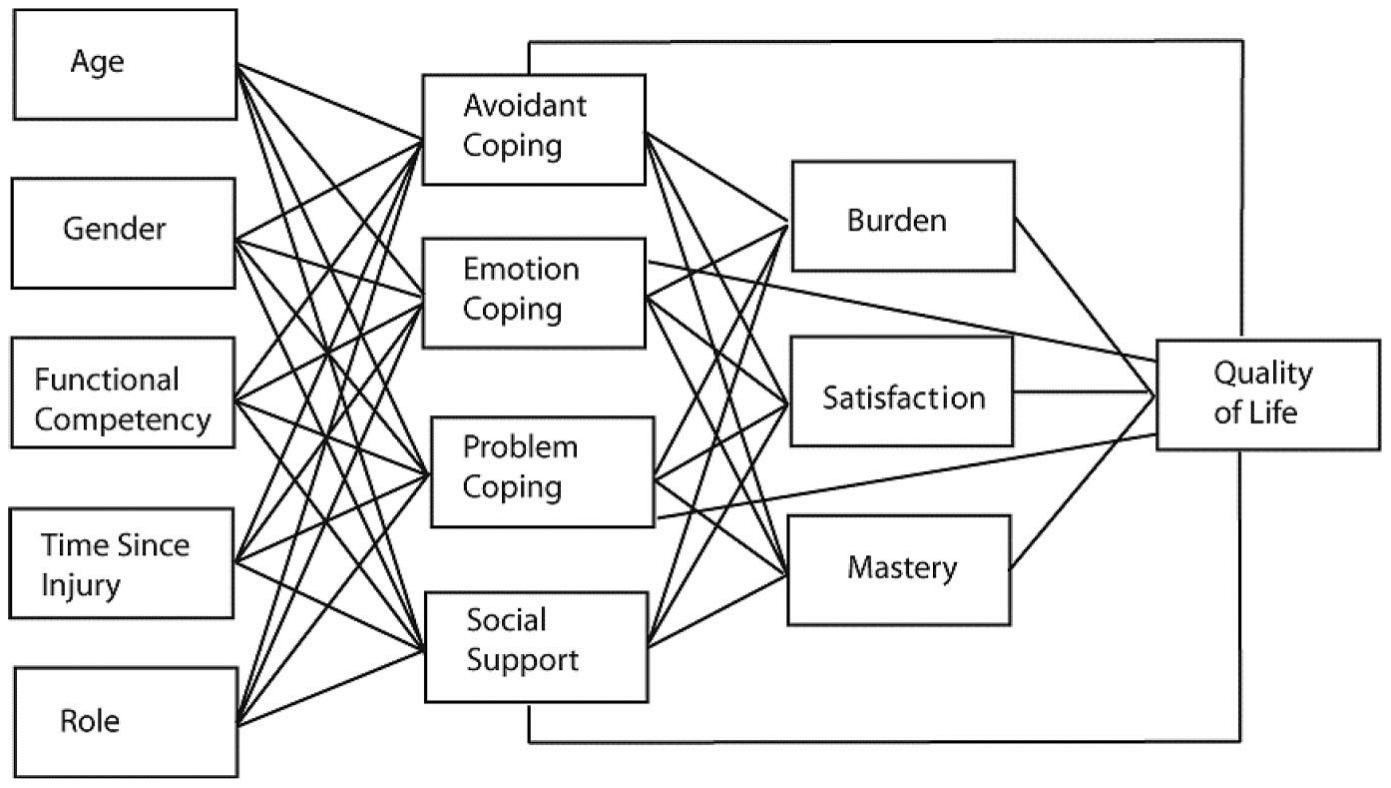
Stress Process Model of Caregiving for Individuals with Traumatic Brain Injury. This figure was reprinted with permission from [15].

The SPMC focuses on the strong association between stress and the high demands of caregiving. This model also considers impacting factors such as psychosocial factors (e.g.: coping and support) and demographic factors (e.g.: age and gender) to assess the caregiver's level of stress as well as potential resiliency factors [15]. Additionally, caregiver quality of life (QOL) and stress appraisal are affected by family needs and social support and the SPMC highlights these factors [21]. Therefore, when a caregiver has a perceived social support network and their important social needs are met, it can directly affect the relationship between caregiver QOL and burden [21]. Ultimately, the SPMC emphasizes the connection between personal and psychosocial resources and how they can affect evaluations of stress outcomes differently [15]. Figure 1 illustrates the stress process model of caregiving for individuals with TBI [15].

### Current Study

According to the Well Florida Council, over 210,000 people in Florida are currently living with a TBI-related disability [27]–[29]. By 2020, this number is expected to increase to nearly 260,000 [27]–[29]. The Brain Injury Association of Florida, Inc. (BIAF) commissioned WellFlorida Council (WFC) to conduct a needs assessment of caregivers of persons with a traumatic brain injury (TBI). The needs assessment was part of BIAF's ongoing work to define the magnitude and pervasiveness of TBI throughout Florida and to augment, improve, refine, and where necessary, create the services to enhance the quality of life for survivors of TBI, their families, and caregivers. The purpose behind the development of the needs assessment is two-fold: (1) to acquire new knowledge about the impact that caring for individuals with TBI has on family members who are caregivers, and (2) to identify the critical resource and supports needs of these families in Florida.

The widely used and empirically supported Stress Process Model of Caregiving (SPMC) guided this study of caregiver needs for persons with TBI. The SPMC model was chosen because it includes a comprehensive conceptualization of caregiving stress appraisal and the inclusion of relevant psychosocial and demographic variables derived from prior research and theory [22]. Additionally, there is a strong association between stress and the high demands of caregiving and the authors were interested in capturing this relationship as it pertains to the caregiver's quality of life (QOL). The survey considers the psychosocial factors that influence caregiver's perceived burden, mastery and satisfaction and the impact caregiving has on the QOL of caregivers and their families.

One integral component of the needs assessment identifies the stages of caregiving and their corresponding needs. The SPMC model serves as a theoretical framework for understanding the stages of caregiving as the SPMC addresses the transitional experiences associated with caregiving by acknowledging the transitional events that take place throughout the course of a TBI [31]. The SPMC provides both a complex and longitudinal view of the caregiver experience, which helps to anticipate and address caregiver needs [31].

In this article, we present findings from the needs assessment survey administered to caregivers in Florida during 2010–2011. Information on the development and implementation of a statewide caregiver needs assessment survey is also included. Implications for future application, research and practice are discussed.

## Methods

The material presented and the work performed within this project adheres to the ethical standards found in the Code of Professional Ethics for Rehabilitation Counselors. Regardless of the specific task, work settings, research, or technologies used, rehabilitation counselors demonstrate adherence to ethical standards and ensure the standards are vigorously enforced. The CRCC Code of Professional Ethics is available for download from http://www.crccertification.com/.

Research was performed in the community with approval of Brain Injury Association of Florida (BIAF) in collaboration with WellFlorida Council, Inc. BIAF, Inc. is a private, nonprofit 501(c)(3) organization created in 1985. The mission of BIAF is to improve the quality of life for all persons with brain injury and their families by creating a better future through brain injury awareness, prevention, research, education, support services and advocacy. WellFlorida Council, Inc. is a private, nonprofit 501(c) (3) organization created in 1969. It is the State designated local health council for 16 counties in North Central Florida and specializes in health-related consultancy for people and projects throughout Florida. WellFlorida's mission is to forge partnerships in planning, research and service that build healthier communities.

### Participants

The target population for this study was adult caregivers of a family member who has sustained a Traumatic Brain Injury (TBI). For the purpose of this study, participants met the following criteria: 1) 18 years of age or older and 2) have primary caregiving responsibilities of a family member with TBI. Using a purposive convenience sample, a total of 53 caregivers were successfully recruited for this study.

### Research Design

This study employed a correlational, non-experimental research design. Participants completed a needs assessment survey questionnaire (described below) that contained questions about the needs of caregivers across the various stages of the caregiving process. Also included in the study are demographic and socioeconomic questions about the relationship between the caregiver and the survivor. Further analyses were conducted to examine if there were differences between caregiver needs, demographic and socioeconomic characteristics, and the stage of caregiving.

### Caregiver Needs Assessment Survey

Caregiver needs were assessed using the 2011 BIAF Caregiver Needs Assessment Survey (BIAF CNAS) developed though commission of the Well Florida Council, Inc. Because this was intended to be administered to the community, several similar surveys were used as guides to develop the BIAF CNAS questions (e.g., 2008 Minnesota Behavioral Risk Factor Surveillance Survey, 2010 Nebraska TBI Caregiver Survey, North Dakota TBI Needs Assessment). Although the surveys used as guides to create the BIAF CNAS are not necessarily validated research questionnaires, the survey intended to uniquely contribute to the literature by determining the needs of caregivers at various stages of the caregiving process. Therefore, the BIAF CNAS represents a culmination of questions that were appropriate for a community based survey to examine factors related to caregivers of TBI survivors such as support and self-reported stress. The BIAF CNAS is a 37-item instrument containing open and closed-ended survey questions about the caregiver and the individual with TBI utilizing a “stages” model of caregiving. The survey instrument consists of a core module including demographic and socioeconomic questions about the caregiver and the survivor of TBI. It also includes a secondary module with questions about the respondents identified stage of caregiving. Incorporation of the secondary module allows for the detection of variation in responses on the basis of the stage of caregiving that the respondent identifies with as well as the standard possible covariates (e.g. age, gender, and ethnicity). Stages of caregiving were incorporated to allow for between-group comparisons that will likely yield valuable information about the needs of specific caregivers and the caregiving process.

The survey includes specific questions about the nature and the severity of the injury, the symptoms of the injury, the accessibility of appropriate healthcare and support services, type of assistance and benefits received, and the nature of the relationship between the caregiver and survivor relevant to the TBI caregiver experience. Caregivers were asked to identify their most pressing needs and concerns, to provide information about the most beneficial resources and supports, and to identify supports that they receive from others.

### Demographic Information

Basic information pertaining to the family unit was collected to provide descriptive information about the family. Specific questions that were asked included information about the caregiver and the family member with TBI. The descriptive information questions pertaining to the caregiver asked basic demographic information including: gender, age, marital status, ethnicity, educational level, employment status, relationship of the caregiver to the person with TBI, and living arrangement (with or without the survivor). Specific questions asked regarding the survivor included: gender, marital status, ethnicity, educational level, employment status, age at the time of the TBI, experienced a TBI more than once, type of injury sustained, and residence.

### Services and Caregiver Strain Information

General information about the strains and services available to the caregiver and survivor were also collected. Information about the caregivers included: support received from other friends or family members, participation in support groups, number of hours spent caring for the survivor, number of days per week providing care for the survivor, changes in daily life due to becoming a caregiver, relationship strain due to caregiving responsibilities, most important concerns, and resources they would benefit from most at this time. Information collected about the TBI survivor included: any benefits received, cognitive or psychological symptoms experienced, support from other sources other than the primary caregiver, and participation in support groups.

### Stages of Caregiving

Stages of caregiving emerged from focus groups conducted in 2010 in the State of Florida. Themes that appeared from the focus groups lead to the development of and were incorporated into the BIAF CNAS. Respondents were asked to identify the stage of caregiving that seemed most relevant to their current situation. *Golden hour/inpatient hospitalization* - This stage encompasses the time from the moment the caregiver learns of the injury to the time that the survivor is discharged from the hospital. *Initial re-entry/settling into home* - The second stage of the caregiving process where the survivor has been discharged from the hospital and the caregiver must now look after their daily needs. *Re-integrating into regular activities* - The third stage of the caregiving process where it becomes important to restore some normality to the extent possible in the lives of the caregiver and survivor. *Surviving long-term* – In this final stage of the caregiving process, the situation has stabilized and now the caregiver must think about the long-term ramifications of the injury and the provision of long-term needs for the survivor.

### Data Collection Procedures

Families with members with TBI were approached through the Brain Injury Association of Florida's (BIAF) monthly electronic newsletter and in-person at the Annual Jamboree and Family Forum in 2011. BIAF is a State affiliate of the Brain Injury Association of American and serves individuals with TBI, their families, caregivers, and professionals throughout the State of Florida. The Annual Jamboree and Family Forum is an annual weekend retreat for TBI survivors and their families. Caregivers were identified through the BIAF database and through partner organizations. Participants were given the opportunity to choose an electronic or paper-based survey format, each containing an identical set of questions. Respondents who chose the web-based format received an email link to the appropriate website, and those who preferred the paper-based method received their survey by mail with a postage paid return envelope. Paper-based responses were secondarily entered into the web-based format by research staff in a secure database. When the caregiver completed the survey, they mailed it, faxed it, or scanned and e-mailed the survey to the researcher. Surveys were examined and analyzed at the time of online submission or when received in the mail. The researcher excluded missing data for the specific analysis pertaining to the missing cases.

### Data Analysis

Web-based survey programs allowed researchers to generate summary reports of the descriptive data collected. Further analyses were performed using statistical software, SPSS 19.0. All analyses were 2-tailed and significant findings were those with p-values less than .05. In addition to descriptive statistics, the research team, in collaboration with the University of South Florida, Policy and Services Research Data Center, ran cross-tabulations and t-tests to compare caregiver needs with demographic and socioeconomic characteristics, along with the stages of caregiving. Identifying these relationships and tracking them over time will help BIAF and partner service delivery organizations to target specific caregivers who have needs for specific types of information and resources.

### Behavioral Therapy Needed Analysis

Independent samples t-tests analyses were performed to analyze difference scores comparing the most beneficial and needed resources and services identified by the caregiver with the number of TBI symptoms experienced by the survivor of TBI (continuous variable). Specifically, a t-test analysis examined if caregivers who reported behavior therapy as the most beneficial and needed resource differed on the reported number of TBI symptoms experienced by the survivor, relative to caregivers who did not report behavior therapy as the most beneficial and needed resource.

#### Stages of Caregiving, Recovery, and Concerns Analysis

To gain a better understanding of the association of symptomology of individuals with TBI with resources and supports needed, chi-square analyses were conducted to determine if there was a relationship between the identified stage of caregiving and the caregiver's most pressing concern. Several pressing concerns associated caring for persons with TBI were examined (all dichotomous): (a) cost of hospital care; (b) experiencing a major life change in daily routine life consequential of TBI; and (c) cost of long-term care. A chi-square analysis also examined the relationship between receiving benefits (medical, financial, entitlements) or not (dichotomous) and the most pressing concerns endorsed by the caregiver. In addition, a chi-square analysis examined the relationship between the time from receiving the injury and the identified stage of recovery.

### Type of Concern, Number of Benefits and Number of Symptoms Analysis

T-test analyses were conducted to examine the differences between the number of benefits received (medical, financial, entitlements) and each type of pressing concern identified by the caregiver. In particular, differences were analyzed between caregivers who believed that hospital care was more important as opposed to those who did not and the likelihood of receiving certain benefits or assistance. Finally, a t-test analysis was computed to examine the difference between TBI caregivers who endorsed making changes in time spent with family in their daily lives vs. those who did not endorse changing family time with the number of TBI related symptoms experienced by the survivor.

## Results

### Descriptive Information for the Sample Population

#### Caregiver and Survivor Demographics

The sample consisted of 53 caregivers. Caregiver ages ranged from 26–81 years old with a mean of 57.9 years (*SD* = 12.05). Caregivers also reported on the demographic characteristics of the individual with a TBI. Therefore, the sample consisted of 53 survivors. The age that the survivor experienced the TBI ranged from 4–81 years old with a mean of 30.3 years (*SD* = 16.67). Table 1 displays the frequency results of demographic information for caregivers and survivors.

**Table 1:** Caregiver and TBI Survivor Demographic Characteristics (N = 53).

Characteristic	Caregiver N (%)	Survivor N (%)
**Gender**		
Male	6 (11.3)	37 (69.8)
Female	46 (86.8)	13 (24.5)
Decline	1 (1.9)	3 (5.7)
**Race/Ethnicity**		
White/Caucasian	44 (83.0)	43 (81.1)
Black/African American	1 (1.9)	1 (1.9)
Hispanic/Latino(a)	4 (7.5)	3 (5.7)
Native American/American Indian	2 (3.8)	1 (1.9)
Asian	0 (0.0)	1 (1.9)
Decline	2 (3.8)	3 (5.7)
**Marital Status**		
Single	3 (5.7)	32 (60.4)
Married	32 (60.4)	10 (18.9)
Divorced	11 (20.8)	5 (9.4)
Widowed	4 (7.5)	0 (0.0)
Other	1 (1.9)	2 (3.8)
Decline	2 (3.8)	3 (5.7)
**Educational Level**		
Less than high school	0 (0.0)	4 (7.5)
High school diploma/GED	10 (18.9)	16 (30.2)
Some college	12 (22.6)	9 (17.0)
Associate's degree	7 (13.2)	10 (18.9)
Bachelor's degree	14 (26.4)	6 (11.3)
Graduate/professional degree	7 (13.2)	2 (3.8)
Decline	3 (5.7)	1 (1.9)
**Employment Status**		
Full-time	17 (32.1)	3 (5.7)
Part-time	5 (9.4)	3 (5.7)
Homemaker	1 (1.9)	0 (0.0)
Unemployed/seeking work	4 (7.5)	4 (7.5)
Unemployed/NOT seeking work	0 (0.0)	18 (34.0)
Retired	16 (30.2)	0 (0.0)
Other	9 (17.0)	21 (39.6)
Decline	1 (1.9)	4 (7.5)
**Relationship to Survivor**		
Parent	31 (58.5)	N/A
Spouse	9 (17.0)	N/A
Child	2 (3.8)	N/A
Sibling	2 (3.8)	N/A
Legal Guardian	1 (1.9)	N/A
Other	5 (9.4)	N/A
Decline	3 (5.7)	N/A

#### Survivor Symptoms Reported

Caregivers reported that the survivors of TBI experienced a wide range of symptoms related to sustaining their injuries. Specifically, the number of symptoms endorsed ranged from 0–14 symptoms, and on average caregivers reported that the survivor experienced 6.2 (*SD* = 3.6) symptoms. Results of symptoms reported from the survey are shown in Table 2 and indicate that there are many commonly reported areas in which survivors of TBI experienced symptoms.

**Table 2:** TBI Survivor Living Arrangement, Etiology and Reported Physical, Psychological, Cognitive and Psychosocial Symptoms (N = 53).

Characteristic	N (%)
**Current Living Arrangement**	
House	28 (52.8)
Apartment	11 (20.8)
Hospital	2 (3.8)
Group home	1 (1.9)
Nursing home	1 (1.9)
Decline	10 (18.9)
**Cause of TBI**	
Motor vehicle crash	29 (54.7)
Fall	4 (7.5)
Pedestrian injury	4 (7.5)
Assault or abuse	3 (5.7)
Decline	11 (20.8)
**TBI Symptoms Reported**	
Attention	32 (60.4)
Short-term Memory	42 (79.2)
Long-term Memory	11 (20.8)
Motor skills	31 (58.5)
Language skills	28 (52.8)
Self-control	19 (35.8)
Aggression	15 (28.3)
Impulsivity	23 (43.4)
Compulsivity	16 (30.2)
Denial	16 (30.2)
Depression	29 (54.7)
Anxiety	23 (43.4)
Insomnia	17 (32.1)
Limited physical mobility	27 (50.9)
Other	5(9.4)

*Note*: Some Caregivers reported that survivors experienced multiple TBI symptoms.

#### Survivor Resources and Support

Caregivers were asked to indicate the type of assistance and benefits that the survivors of TBI were receiving at the time of the survey. Data are summarized in Table 3 and indicate that most survivors receive some type of assistance and/or benefits from a variety of sources. On average, survivors receive services from approximately 1.98 (SD = 1.32; Range = 0–5) sources.

**Table 3:** Type of Assistance, Benefits and Caregiver and TBI Survivor Support Systems (N = 53).

Type of Assistance/Benefits	N (%)
Medicare	20 (37.7)
Medicaid	19 (35.8)
Social Security	11 (20.8)
Social Security Disability	31 (58.5)
Veteran's Disability	1 (1.9)
Legal Settlement	0 (0.0)
Unemployment insurance	0 (0.0)
Worker's Compensation	2 (3.8)
Food stamps	11 (20.8)
Private insurance	10 (18.9)
**Survivor support from others**	N (%)
Spouse	10 (18.9)
Sibling	17 (32.1)
Parent	22 (41.5)
Child	7 (13.2)
Significant other	0 (0.0)
Friend	8 (15.1)
Caregiver	6 (11.3)
Legal guardian	0 (0.0)
**Caregiver support from others**	N (%)
Spouse	17 (32.1)
Sibling	5 (9.4)
Parent	8 (15.1)
Child	9 (17.0)
Significant other	2 (3.8)
Friend	9 (17.0)
Caregiver	4 (7.5)
Legal guardian	1 (1.9)
**Resources and support**	N (%)
Caregiver participation in support group	18 (34.0)
Survivor participation in support group	24 (45.3)

Table 3 also displays the results of the different type of people who provide care and support for survivors. Results suggest that within this particular sample, most caregiving responsibilities fall upon the immediate family members of the survivor. Support from others for the survivor ranged from 0–3 people, with a mean of 1.30 (SD = .92) people who provide support to the survivor. Caregiver support received from others was reported with the average amount of support being provided by one other person (SD = 1.04; Range = 0–4). Caregivers were most likely to receive support from their spouses if they were married. Sixty percent of the caregivers included in this study were married. Those who were not married received support from parents, friends, children, siblings and other caregivers.

A supplementary question asked whether caregivers or survivors participated in support groups (additional resources and supports). Caregivers indicated that slightly less than half, or 45.3% (N = 24), of survivors belonged to support groups. Among caregivers themselves, only 34% (N = 18) said that they participated in support groups. The probe for this particular question “why or why not?” elicited a variety of different responses, some of which suggested that caregivers placed the needs of the survivor over their own need for support. Other responses indicated that caregivers were not aware of support groups in their areas, or were skeptical about the benefit they could gain by participating in such groups.

#### Caregiver Challenges, Concerns, and Needs

Table 4 displays results of changes to the caregiver's life, strains to the caregiver's relationships, and the most important concerns according to the caregiver. Another challenge to caregivers is maintenance of social relationships with others amidst their caregiving duties. The survey asked caregivers to identify relationships that had been strained as a result of becoming caregivers. These results are also displayed in Table 4. The final questions of the survey assessed the caregiver's most pressing needs and concerns related to the family member with TBI. These needs and concerns encompass present and future caregiving needs and concerns related to sustaining and living with current and long-term disability consequential of TBI. Results are also displayed in Table 4.

**Table 4:** Changes to Daily Life because of Caregiving, Relationship Strain, and Most Pressing Concerns among Caregivers.

Changes to caregiver's daily life because of caregiving	N (%)
Work schedule	29 (54.7)
Sleep schedule	21 (39.6)
School schedule	1 (1.9)
Family time	28 (52.8)
Personal leisure time	42 (79.2)
Child care	4 (7.5)
Housing	22 (41.5)
Transportation	28 (52.8)
Personal healthcare	23 (43.4)
Church/religious activities	15 (28.3)
**Caregiving responsibilities strain relationships with**	**N (%)**
Survivor	22 (41.5)
Parents	3 (5.7)
Children	20 (37.7)
Spouse	23 (43.4)
Significant other	3 (5.7)
Siblings	6 (11.3)
Relatives by marriage	3 (5.7)
Co-workers	5 (9.4)
Neighbors	7 (13.2)
Friends	16 (30.2)
**Most pressing concerns of caregivers**	**N (%)**
Severity of the injury	9 (17.0)
Prognosis (likelihood of recovery)	0 (0.0)
Cost of hospital care	7 (13.2)
Implication for major change in the survivor's life	24 (45.3)
Implication for major change in your life	18 (34.0)
Cost of long-term care	13 (24.5)
Survivor's need for long-term material, emotional, and social support	34 (64.2)
Your need for long-term material, emotional, and social support	21 (39.6)
Survivor's need for round the clock support/supervision from you	21 (39.6)

Caregivers identified resources that they felt were most beneficial and necessary when providing care. Results are displayed in Table 5. Twenty caregivers (37.7%) indicated that the most beneficial resource was a patient or caregiver advocate, followed by a wider network of support from friends and family (N = 18; 34.0%), and behavioral therapy for the survivor (N = 15; 28.3%).

**Table 5:** Most Important Needs According to Caregivers.

Most beneficial/needed resources	N (%)
Patience skills training	11 (20.8)
A wider network of support, e.g. friends, family	18 (34.0)
Support group of fellow caregiver	11 (20.8)
Trustworthy respite care	14 (26.4)
A patient/caregiver advocate	20 (37.7)
Behavioral therapy for the survivor	15 (28.3)
Sensitivity training for health care providers	8 (15.1)
A guide/glossary for TBI related medical terminology	6 (11.3)
Other	7(13.2)

#### *Note*: Some Caregivers selected more than one most beneficial/needed resource.Stage of Caregiving

Caregivers were asked to identify the stage of caregiving which they felt best reflected their current caregiving situation. Survey respondents choose one of the following, previously outlined stages: (a) initial injury/inpatient hospitalization (N = 1; 1.9%), (b) re-entry/settling into home (N = 2, 3.8%), (c) reintegration (N = 9, 17.0%), and (d) surviving long-term (N = 34, 13.2%). The largest number of caregivers reported that they were in the “surviving the long-term” stage of caregiving, indicating that the majority was in the final stage of the caregiving process.

### Group Comparisons

#### Behavioral Therapy Needed

T-test results indicated that caregivers who reported behavior therapy as a most beneficial and needed resource had significantly higher reports of number of symptoms experienced by the survivor compared to caregivers who did not indicate behavior therapy as a most needed and beneficial resource [*M* = 9.13 symptoms (*SD* = 2.53) vs. *M* = 5.05 (*SD* = 3.38), *t* = 4.22, df = 51, *p*<.05]. Further, Cohen's effect size value (*d* = 1.18) indicated a large effect size.

#### Stages of Caregiving, Recovery, and Concerns

Chi-square results revealed significant differences between the stage of caregiving, stage of recovery, and concerns reported by the caregiver. Findings indicated that the cost of hospital care (*X* ^2^ =  8.87, df = 3, *p*<.05, Ф = .46), experiencing a major life change (*X* ^2^ = 13.07, df = 3, *p*<.05, Ф = .53), and the cost of long-term care (*X* ^2^ = 12.36, df = 3, *p*<.05, Ф = .50), are pressing concerns that vary for caregivers of individuals with TBI based on the stage of caregiving. Caregivers in the *Golden hour/inpatient* hospitalization stage indicated that cost of hospital care and cost of long-term care were pressing concerns. Caregivers in the *Initial re-entry/settling* stage reported that experiencing a major life change and the cost of long-term care were pressing concerns. Caregivers in the *Re-integrating into regular activities* indicated that experiencing a major life change was a pressing concern. Caregivers in the *Surviving long-term* stage reported that hospital costs and long-term costs of care were not pressing concerns and they were not experiencing major life changes.

There was also a significant difference between benefits and most pressing concerns. Because one cell had an expected count of less than 5, Fisher's exact statistic was interpreted (Fisher's exact statistic = .04, *p*<.05, Ф = .34). Specifically, those with no benefits were more likely than those with at least one benefit to list cost of hospital care as a pressing concern (42.9% vs. 8.7%). Finally, there was a trend that those with more time from injury were more likely to be in the long-term survival stage (*X* ^2^ = 16.43, df = 9, *p*<.10, Ф = .06).

#### Type of Concern, Number of Benefits and Number of Symptoms

T-test results comparing the type of concerns and number of benefits showed that those who endorsed cost of hospital care as a pressing concern had significantly less benefits/assistance [*M* = 0.86 benefits, (*SD* = 1.07) vs. *M* = 2.15 benefits, (*SD* = 1.28) *t* = −2.54 (54), *p*<.05]. Further, Cohen's effect size value (*d* = .69) indicated a medium effect size. This indicates that caregivers who believed that hospital care was more important were less likely to receive benefits or assistance, relative to those who are less likely to believe that hospital care is important. There was a trend toward significance that those who endorsed changing family time had more symptoms than those who did not change family time [*M* = 7.11 benefits, (*SD* = 3.63) vs. *M* = 5.20 benefits, (*SD* = 3.46) *t* = 1.95 (51), *p*<.10]. Further, Cohen's effect size value (*d* = .55) indicated a medium effect size.

## Discussion

In this study, the Stress Process Model of Caregiving (SPMC) served as a theoretical framework for assessing the needs of caregivers of individuals with TBI. Because the SPMC examines both the positive and negative aspects of the caregiver experience, the needs assessment survey utilized in this study inquired about caregiver supports and resources as well as strains and concerns. Additionally, within these categories, the SPMC helps to evaluate impacting factors such as psychosocial factors and demographic factors. Findings of this study, through the evaluation of needs in the respective stages of caregiving, reinforce many of the transitional needs highlighted in the SPMC. Caregiver needs continue to evolve throughout the caregiving process.

The data in this study, as well as in previous studies, show a link between challenging survivor injuries, the amount of time spent caregiving and increased caregiver emotional and physical distress [18], [23], [26]. Second, findings in this study highlight the survivor and caregiver characteristics while identifying the stage of caregiving. Previous studies mirror the significance of caregiving stages, as well as the transition of caregiver needs as the person with TBI progresses from acute hospital care to long-term in-home care [7], [18]. This is significant because, as the data shows, caregiver needs are often related to their stage of caregiving. For example, individuals with a longer time from injury are more likely to be in the surviving long-term or final stage of caregiving with concerns and needs such as long-term material, social and financial support for the caregiver and individual with TBI as opposed to someone in the initial injury/inpatient hospitalization or first stage of caregiving, with needs such as education about TBI and affordable/accessible medical care. Many caregivers in the surviving long-term stage reported that hospital and long-term costs of care were not pressing concerns and they were not experiencing major life changes. These findings are similar to other studies that found that a majority of caregivers did not report caregiving stress or strain when providing care for persons with TBI [34]–[38]. There may be many reasons for the underreporting of concerns or strain including adaptation to the caregiving role over time, better use of coping strategies, and many caregivers may mask feelings of strain or concern. Further, caregivers may be influenced though feelings of guilt or sense of duty, which may affect their response to the survey questionnaire [38].

Finally, findings in our study illuminate the importance of caregiver knowledge and understanding of benefits and services available for survivors of TBI and their caregivers, as well as services to assist caregivers with long-term physical, emotional, financial, social and informational support needs. Caregivers indicated that the most beneficial services would be a caregiver advocate, wider network of support for family and friends and behavioral therapy for the survivor. These results demonstrate the most pressing unmet caregiver needs in the surveyed population. This is substantiated in previous studies, which emphasize that survivors of TBI receive better care when their caregiver perceives sufficient social support, including adequate community services [8], [25]. This improved understanding will help researchers and practitioners better address the holistic life-long needs of caregivers and their families.

### Limitations of the Study

Limitations of the study include a relatively small sample size which should be taken into consideration when interpreting the results. A larger and more representative sample is needed to contribute to the validity, reliability and significance associated with these findings. Also, the sample consisted predominantly of Caucasian females who have post-high school educational training. Further, a majority of caregivers were in the surviving the long-term stage of caregiving. This sample was unrepresentative of the various cultures and genders that experience and care for individuals who sustain a TBI. An additional limitation is that the survey instrument has not been tested and does not include any baseline measures. Future research should consider creating a survey based on formal measures to examine quality of life, burden, and perceived stressed. Finally, participation in the study was advertised only to current and former members of BIAF, in TBI support groups recognized by BIAF, and at the BIAF's Jamboree and Family Forum. Caregivers who are not active members of BIAF, who do not attend support groups, and who do not participate in BIAF sponsored events were not represented. Future recruitment efforts should include mechanisms to expand access to a more representative sample that can, in turn, be generalized to the population of caregivers of individuals with TBI.

## Conclusions

The present study provides additional support for the utility of applying the Stress Process Model of Caregiving for Individuals with TBI in the context of caring [15], [21]. A caregiver needs assessment, based on this model of caregiving, allowed for identification of caregiver needs in Florida. This identification fosters a better understanding of the multiple barriers and challenges experienced by caregivers across the stages of caregiving. Although this study was limited by the number of participants, significant differences were found between the stage of caregiving, stage of recovery and pressing concerns reported by the caregiver, which indicate that caregiving and related family needs are ever-present and continue to change over time. Critical resource and supports, including long-term social, emotional, educational, informational and financial needs were highlighted by caregivers of individuals with TBI in Florida. These findings illustrate the importance of following caregivers of individuals with TBI after discharge from acute care. However, findings also highlight that a large number of caregivers may not report needs or concerns when providing care for persons with TBI [38].

Study findings provide a unique perspective of the present needs and future concerns of caregivers of individuals with TBI and point to several directions for future research and practice. The caregivers of individuals with TBI sampled in this survey and the survivors they care for face many challenges. The complexity of the injuries, which can be physically as well as emotionally and psychologically exhausting, are further complicated by limited material, financial, and social resources for both the caregiver and the survivor. Future research is needed for validation of the caregiver needs assessment survey as a potential instrument for understanding and addressing the complex resource and support needs of caregivers of individuals with TBI and their families. Finally, incorporating this understanding may influence and guide the development, implementation and modification of timely and cost-effective interventions and treatment programs designed to support the unique caregiving and familial needs and challenges experienced by caregivers of individuals with disabilities consequential of TBI.
